# Teaching Men How to Live

**Published:** 1906-07-07

**Authors:** 


### TEACHING MEN HOW TO LIVE.

Mr. James Cantlie, speaking at the commemoration-day proceedings at Livingstone College emphasised the very great importance of training in elementary medicine, surgery, and hygiene to missionaries going to uncivilised lands. Many missionaries, he said, went out to die for Christianity, but he considered it a nobler aim to live for Christianity, and the College had been established for the purpose of teaching men how to live. He compared the course of training to the St. John's Ambulance course, and showed that whilst in England accidents were a very frequent occurrence, there were very few such accidents in lands where there were no mines, manufactories, or wheeled vehicles. In tropical countries, however, disease struck down its victims with terrible suddenness, and it was necessary that ordinary missionaries should know how to deal with the most common diseases. People said that a little knowledge was a dangerous thing, but, like the teaching of the St. John's Ambulance, the course at Livingstone College was complete of its kind, and it gave to missionaries exactly the teaching which they required in medical subjects, and he considered that no missionary should go abroad without some such training. He regretted that the chief missionary societies had not taken the matter up in the way that they should do, and he considered that many of them were trading on the religious fervour of their representatives through sending them out without proper equipment in the way of medical knowledge, and in so doing he considered that they were juggling with men's lives.

